# Supplementary material for: The Systems Biology Research Tool: evolvable open-source software
Source: BMC Syst Biol. 2008 Jun 29;2:55. doi: 10.1186/1752-0509-2-55 (PMC2446383; doi:10.1186/1752-0509-2-55)
Supplement: Additional file 1 — SBRT Archive. An archive of the current version of the Systems Biology Research Tool. [file 1752-0509-2-55-S1.zip › sbrt-1.4.0/doc/developers_guide/api/sbrt/kernel/fba/proc/CvFbaOptProcess.html]

CvFbaOptProcess


|  |  |  |
| --- | --- | --- |
| **Overview** \| **Package** \| **Class** \| **Use** \| **Tree** \| **Deprecated** \| **Index** \| **Help** | |  |
| **PREV CLASS**   **NEXT CLASS** | **FRAMES**    **NO FRAMES**     **All Classes** |  |
| SUMMARY: NESTED \| FIELD \| CONSTR \| METHOD | DETAIL: FIELD \| CONSTR \| METHOD |  |


---


## sbrt.kernel.fba.proc Class CvFbaOptProcess<R>

```
java.lang.Object
  sbrt.kernel.fba.proc.FbaProcess<R>
      sbrt.kernel.fba.proc.CvFbaOptProcess<R>
```

**Type Parameters:**: `R` - the results type.

**All Implemented Interfaces:**: KernelProcess<R>

**Direct Known Subclasses:**: CvFluxIntervalProcess, SvCdHarFluxVectorProcess, SvInitialPointProcess

---

``` public abstract class CvFbaOptProcess<R> extends FbaProcess<R> ```

This class is used to represent constraint-variation Flux Balance Analysis
experiments. It offers two static methods to aid in this sort of experiment.

**Author:**
:   This class was written and documented by Jeremiah Wright
    while in the Wagner lab.

---

**Constructor Summary**

`CvFbaOptProcess()`


| **Method Summary** | |
| --- | --- |
| `static void` | `restoreConstraints(FluxConstraints constraints, FluxCvComputer computer)`             Restores the provided constraints in the provided constraint-variation computer. |
| `static void` | `setConstraints(FluxConstraints constraints, FluxCvComputer computer)`             Sets the provided constraints in the provided constraint-variation computer. |

**Methods inherited from class sbrt.kernel.fba.proc.FbaProcess**

`getFluxome, toString`

**Methods inherited from class java.lang.Object**

`clone, equals, finalize, getClass, hashCode, notify, notifyAll, wait, wait, wait`

**Methods inherited from interface sbrt.kernel.KernelProcess**

`run`

**Constructor Detail**

### CvFbaOptProcess

```
public CvFbaOptProcess()
```


**Method Detail**

### setConstraints

```
public static void setConstraints(FluxConstraints constraints,
                                  FluxCvComputer computer)
```

:   Sets the provided constraints in the provided constraint-variation
    computer.

    :   **Parameters:**: `constraints` - the set of flux constraints.: `computer` - the computer in which to set the provided constraints.

---


### restoreConstraints

```
public static void restoreConstraints(FluxConstraints constraints,
                                      FluxCvComputer computer)
```

:   Restores the provided constraints in the provided constraint-variation
    computer.

    :   **Parameters:**: `constraints` - the set of flux constraints to be restored.: `computer` - the computer in which to restore the provided constraints.


---


|  |  |  |
| --- | --- | --- |
| **Overview** \| **Package** \| **Class** \| **Use** \| **Tree** \| **Deprecated** \| **Index** \| **Help** | |  |
| **PREV CLASS**   **NEXT CLASS** | **FRAMES**    **NO FRAMES**     **All Classes** |  |
| SUMMARY: NESTED \| FIELD \| CONSTR \| METHOD | DETAIL: FIELD \| CONSTR \| METHOD |  |


---
